# Risk of incident cardiovascular disease events among older Asian, Native Hawaiian, and Pacific Islander colorectal cancer survivors in the United States: a cohort study

**DOI:** 10.1186/s40959-025-00440-4

**Published:** 2026-01-12

**Authors:** Timothy Nguyen, Chun-Pin Esther Chang, Randa Tao, Kuangyu Liu, Zuo-Feng Zhang, Mia Hashibe

**Affiliations:** 1 Department of Epidemiology, School of Public Health, UCLA Fielding, University of California Los Angeles, Los Angeles, CA USA; 2 Division of Public Health, Department of Family & Preventive Medicine, University of Utah School of Medicine, Salt Lake City, Utah, USA; 3 Huntsman Cancer Institute, Salt Lake City, Utah, USA; 4 Department of Radiation Oncology, University of Utah School of Medicine, Salt Lake City, Utah, USA; 5 Mayo Clinic, Phoenix, AZ USA

**Keywords:** Colorectal cancer, Cardiovascular diseases, Asian, Hawaiian, And Native Pacific Islanders

## Abstract

**Background:**

Among Asian, Native Hawaiian, and Pacific Islanders (ANHPI) in the United States, cancer and cardiovascular disease are the leading causes of death. Colorectal cancer (CRC) is the third most common cancer among ANHPIs, with improving survival rates. However, the risk of cardiovascular disease (CVD) events among ANHPI CRC survivors is unknown, especially within disaggregated ANHPI race and ethnicity groups.

**Methods:**

We estimated the risk of CVD events among ANHPI CRC survivors within the SEER-Medicare database. Composite CVD, heart failure, ischemic heart disease, and stroke/transient ischemic attack were identified using International Classification of Diseases (ICD) diagnostic codes. Cox proportional hazard models were used to estimate hazard ratios (HR) and 95% confidence intervals (CI) for CVD events among ANHPI regional subgroups.

**Results:**

Compared to non-Hispanic White (NHW) CRC survivors, the risk of composite CVD was lower among East and Southeast Asian CRC survivors at > 1 years after initial cancer diagnosis. The risk of composite CVD, heart failure, and ischemic heart disease were lower among East and Southeast Asians for follow-up > 5 years. When compared to East Asians, NHW and Southeast Asian CRC survivors had a higher risk of composite CVD, heart failure, and ischemic heart disease where South Asians had a higher risk of ischemic heart disease.

**Conclusions:**

Within the disaggregated ANHPI race and ethnicity groups of CRC survivors, our results support heterogeneity of incident CVD events. Further research is needed to develop public health interventions to address the disparities in CVD risk, especially among the high-risk groups of South Asian and Southeast Asian CRC survivors.

**Supplementary Information:**

The online version contains supplementary material available at 10.1186/s40959-025-00440-4.

## Background

Colorectal cancer (CRC) is the third most common cancer in the United States, with an estimated 152,810 incident cases diagnosed in 2024 [1]. It is the second leading cause of cancer death and has a 5-year relative survival rate of 64%. Advancements in CRC screening and treatments have improved the survival rate over time [2]. With improved lifespans for CRC survivors, cardiovascular disease (CVD) is a rising concern among cancer survivors. CVDs are diseases of the heart and blood vessels, including heart failure, ischemic heart disease, and stroke/transient ischemic attacks [3]. In the United States, CVD is the leading cause of death, with approximately 868,273 deaths in 2022 [4]. CVD is also the leading cause of death among cancer survivors, accounting for more deaths than cancer itself [5]. Compared to the general population, colorectal cancer patients have a higher risk of cardiovascular disease death [6, 7]. There are many overlapping risk factors among CRC and CVD, such as obesity, diabetes mellitus, physical inactivity, unhealthy diet, tobacco use, and alcohol use [4, 8]. Several population-based cohort studies in the US, UK, and Denmark have reported higher CVD risk among cancer survivors compared to the general population [9–11]. In the Atherosclerosis Risk in Communities study, CRC survivors had a higher CVD risk than those without cancer, independent of known CVD factors [11, 12]. A recent systematic review also observed low cardiovascular health and elevated risk of cardiovascular health outcomes among cancer survivors [13]. In cancer patients, tumors release proinflammatory cytokines that may lead to the formation of atherosclerotic plaque and CVD events [14]. Chemotherapy for CRC that consists of fluorouracil or capecitabine can potentially result in cardiovascular toxicity, especially among patients with pre-existing CVD conditions and risk factors [15, 16].

The overlap between CRC and CVD is a growing concern among Asian, Native Hawaiian, and Pacific Islanders (ANHPI), one of the fastest-growing populations in the United States. Among ANHPIs, CRC has the third highest incidence of all cancer for both men and women in the United States [17, 18]. When examining ANHPI race and ethnicity groups and sex, CRC incidence was heterogenous. Between 2016 and 2020, CRC was the leading cancer type for Asian men of Korean, Hmong, and Cambodian descent [19]. CRC also ranked among the most common cancer types for both genders of other Asian racial and ethnic groups including Chinese, Japanese, Filipino, Vietnamese, Laotian, Thai, Asian Indian, and Pakistani and Native Hawaiians, Chamorros, Guamanians, Samoans, Tongans, and Fijians. Alongside cancer, CVD is the other leading cause of death among ANHPI, with heterogeneity between different race and ethnic subgroups.

Compared to other racial groups, ANHPIs have relatively better health outcomes. However, there are differences in health outcome when comparing Asians and NHPIs. Asians have reported lower relative incidence and mortality rates of cancer and heart disease, along with lower prevalence of risk factors such as tobacco use, alcohol use, and obesity [20]. For Native Hawaiian and Pacific Islanders (NHPI), CVD risk on the national level is unclear, but they are more susceptible to stroke due to higher prevalence of obesity, smoking, and hypertension [21, 22]. This highlights an often overlooked issue in racial disparities health research: the aggregation of ANHPI races and ethnicities. The aggregation of ANHPI into a single category masks the health disparities and differing determinants of health experienced by the variety of race and ethnicity subgroups. The ANHPI group consists of over 50 ethnicities with different cultures, socioeconomic disparities, and health disparities [23]. NHPI are often group with Asians due to the lack of data, which obscures their disproportionate and often worse health outcomes.

While there are prior studies examining CVD risk among colorectal cancer survivors, there are no studies that have examined CVD risk among disaggregated ANHPI groups [12, 24]. To examine these racial and ethnic disparities, our study will disaggregate the larger ANHPI group into regional subgroups, including East Asian, Southeast Asian, South Asian, and NHPI. Among these regional subgroups, we hypothesize that older CRC survivors of South Asian and NHPI descent will have a higher risk of incident CVD events compared to the other ANHPI subgroups. In two SEER-Medicare studies for breast cancer and lung cancer, ANHPI cancer survivors were observed to have a lower risk of CVD compared to non-Hispanic Whites (NHW) cancer survivors. However, analysis of disaggregated ANHPI groupings found heterogeneity, with South Asians, Southeast Asians, and Native Hawaiian Pacific Islanders having a higher risk of CVD outcomes compared to East Asian cancer survivors [25, 26]. The aim of our study is to investigate differences in the risk of incident CVD between older ANHPI and NHW CRC survivors in the SEER-Medicare dataset and to assess whether these disparities differ among disaggregated regional subgroup.

## Methods

We conducted a population-based cohort study with CRC survivors identified from the SEER-Medicare linked database from 2000 through 2019. SEER (Surveillance, Epidemiology, and End Results) is a population database that contains demographic, cancer characteristics, and mortality data, which is paired with the Medicare database to link healthcare claims for cancer patients that are Medicare beneficiaries. The Medicare claims include International Classification of Disease, Ninth and Tenth Revision (ICD-9 and ICD-10) diagnostic and procedure codes from in-patient care and hospitalization (Part A), physician/supplier bills (Part B), and outpatient care. We obtained approval for this study from the Institutional Review Board at the University of Utah.

**Fig. 1:**
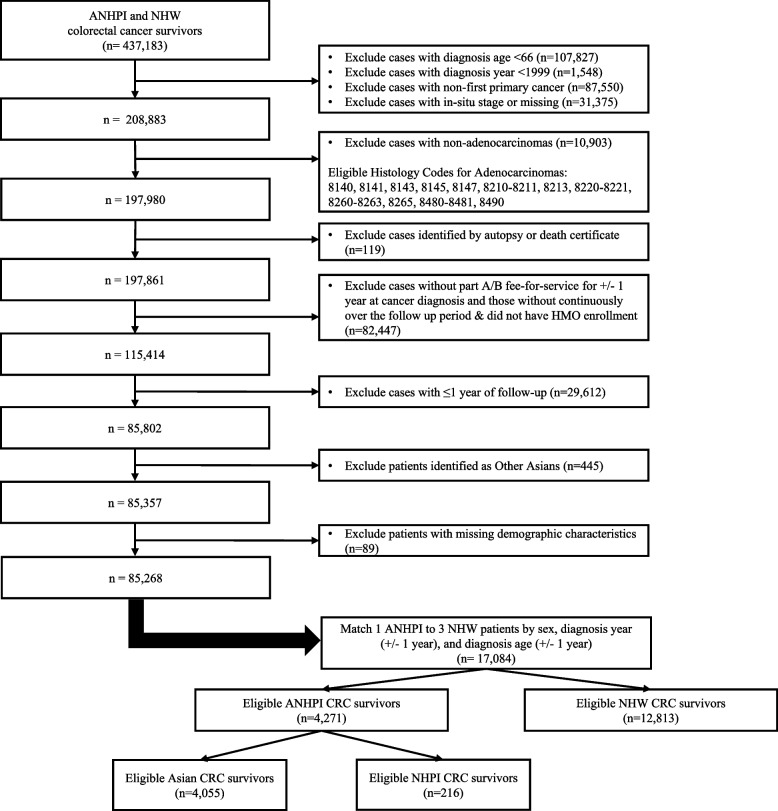
Selection criteria for ANHPI and NHW CRC survivor cohort utilized in this SEER-Medicare study. Abbreviations: ANHPI, Asian, Native Hawaiian, and Pacific Islander; CRC, colorectal cancer; HMO, health maintenance organization; NHW, Non-Hispanic White

CRC survivors were included in this study if they met the following eligibility criteria: 1) were at least 66 years of age, 2) was diagnosed with first primary invasive CRC between 2000 and 2017, 3) had adenocarcinomas (includes histology codes 8140, 8141, 8143, 8145, 8147, 8210–8211, 8213, 8220–8221, 8260–8263, 8265, 8480–8481, 8490), 4) had continuous Medicare Parts A and B coverage throughout the follow-up period, and 5) had no health maintenance organization (HMO) enrollment. Claims for HMO enrollees are not reported to Medicare, and therefore these patients will potentially have missing claims data for the outcome of interest. Patients were excluded if they had less than one year of follow-up, were diagnosed with CRC based on autopsy or death certificate, had missing month of diagnosis, or had unknown stage information. Patients from the Idaho, New York, and Massachusetts SEER registries were also excluded due to missing cancer treatment and death information. International Classification of Diseases for Oncology, 3rd edition (ICD-O-3) codes were used to identify CRC diagnosis (C180-C189 and C260 for colon, C199 and C209 for rectum), excluding cancers of the appendix (C181) and anus (C210-C212 and C218). We matched ANHPI CRC patients with up to three NHW CRC patients by sex, diagnosis year (± 1 year), and age at cancer diagnosis (± 1 year, Fig. 1).

CVD events were identified from Medicare claims using ICD-9 and ICD-10 diagnostic codes (Supplemental Table 1). The diagnosis codes were recategorized as heart failure, ischemic heart disease, and stroke/transient ischemic attack based on categories from the Chronic Conditions Data Warehouse (27 CCW Chronic Conditions Algorithms) [27]. A composite CVD variable was created for those with any of the three CVD events. The composite CVD date was assigned as the earliest date of the three CVD events. Individuals diagnosed with CVD prior to the follow-up time for each period were classified as prevalent cases. A rule-out algorithm was used to confirm CVD diagnoses, and unconfirmed diagnoses were not counted if CVD ICD codes did not appear at least once in hospital claims and at least twice in physician or outpatient claims within 30–60 days.

**Table 1:** Demographic characteristics of NHW, Asian, and NHPI colorectal cancer survivors from SEER-Medicare data, by regional subgroup (*n* = 17,084)

	**NHW (*n* = 12,813)**	**East Asian (*n* = 2772)**	**Southeast Asian (*n* = 1108)**	**South Asian (*n* = 175)**	**NHPI (*n* = 216)**
Age at Cancer Diagnosis					
66 to 70	2613 (20.4)	504 (18.2)	265 (23.9)	47 (26.9)	55 (25.5)
71 to 75	3189 (24.9)	642 (23.2)	298 (26.9)	49 (28.0)	74 (34.3)
76 to 80	3114 (24.3)	710 (25.6)	245 (22.1)	37 (21.1)	46 (21.3)
81 to 100	3897 (30.4)	916 (33.0)	300 (27.1)	42 (24.0)	41 (19.0)
Sex					
Male	6204 (48.4)	1302 (47.0)	559 (50.5)	96 (54.9)	111 (51.4)
Female	6609 (51.6)	1470 (53.0)	549 (49.5)	79 (45.1)	105 (48.6)
Education^a^					
< = 40% had some college education	2288 (17.9)	396 (14.3)	233 (21.0)	12 (6.9)	22 (10.2)
40%- < = 60% had some college education	4622 (36.1)	861 (31.1)	464 (41.9)	38 (21.7)	99 (45.8)
60%- < = 80% had some college education	4196 (32.7)	1104 (39.8)	329 (29.7)	76 (43.4)	79 (36.6)
> 80% had some college education	1707 (13.3)	411 (14.8)	82 (7.4)	49 (28.0)	16 (7.4)
Household Income^a^					
< $50,000	5571 (43.5)	963 (34.7)	386 (34.8)	32 (18.3)	72 (33.3)
$50,000 to < $60,000	2003 (15.6)	395 (14.2)	203 (18.3)	20 (11.4)	38 (17.6)
$60,000 to < $70,000	1537 (12.0)	337 (12.2)	154 (13.9)	25 (14.3)	29 (13.4)
$70,000 +	3702 (28.9)	1077 (38.9)	365 (32.9)	98 (56.0)	77 (35.6)
SEER Registry Region					
West	4404 (34.4)	2569 (92.7)	1006 (90.8)	101 (57.7)	200 (92.6)
South	3384 (26.4)	48 (1.7)	25 (2.3)	16 (9.1)	**
Northeast	3155 (24.6)	129 (4.7)	61 (5.5)	46 (26.3)	**
Midwest	1870 (14.6)	26 (0.9)	16 (1.4)	12 (6.9)	**
Rural Status					
Urban	10,227 (79.8)	2642 (95.3)	1074 (96.9)	> 164 (> 93.7)^c^	171 (79.2)
Rural	2586 (20.2)	130 (4.7)	34 (3.1)	< 11 (< 6.3)^c^	45 (20.8)
Follow-up Time					
> 1–5	6526 (50.9)	1327 (47.9)	658 (59.4)	111 (63.4)	106 (49.1)
> 5 −10	3904 (30.5)	819 (29.5)	279 (25.2)	48 (27.4)	70 (32.4)
> 10	2383 (18.6)	626 (22.6)	171 (15.4)	16 (9.1)	40 (18.5)
Baseline Charlson Comorbidity Index^b^					
0	7425 (57.9)	1438 (51.9)	516 (46.6)	84 (48.0)	113 (52.3)
1	3359 (26.2)	799 (28.8)	338 (30.5)	57 (32.6)	60 (27.8)
2 +	2029 (15.8)	535 (19.3)	254 (22.9)	34 (19.4)	43 (19.9)
Obesity before cancer diagnosis					
No	11,505 (89.8)	2706 (97.6)	1070 (96.6)	> 164 (> 93.7)^c^	191 (88.4)
Yes	1308 (10.2)	66 (2.4)	38 (3.4)	< 11 (< 6.3)^c^	25 (11.6)
Tobacco use disorder before cancer diagnosis					
No	11,873 (92.7)	2694 (97.2)	1067 (96.3)	> 164 (> 93.7)^c^	199 (92.1)
Yes	940 (7.3)	78 (2.8)	41 (3.7)	< 11 (< 6.3)^c^	17 (7.9)

*NHW* Non-Hispanic White, *NHPI* Native Hawaiian and Pacific Islander

^a^Census-tract level data

^b^Charlson Comorbidity Index excluded cardiovascular conditions

^c,**^Cell value coarsened due to CMS cell size suppression policy

Demographic and clinical information was obtained from the SEER data file. These covariates included sex, race and ethnicity, census tract-level education and household income, rural residence, SEER cancer registry, year of cancer diagnosis, age at cancer diagnosis, cancer stage, histology, cancer site, first course of cancer treatment (surgery, radiation therapy, chemotherapy), and vital status. For race and ethnicity, ANHPIs were further classified into regional subgroups. Regional subgroups included East Asian (Chinese, Japanese, and Korean), Southeast Asian (Filipino, Vietnamese, Laotian, Hmong, Kampuchean, and Thai), South Asian (Asian Indian or Pakistani), and NHPI. Finer ANHPI race and ethnicity groups included Chinese, Filipino, Japanese, Vietnamese, Korean, Asian Indian or Pakistani, Native Hawaiian, and Other Pacific Islanders. Other Asians and Asians not otherwise specified were excluded as this subgroup is not well-defined and represents a heterogenous group of unknown Asian race and ethnicities. Medicare claims were used to identify CVD events and comorbidities. Baseline Charlson Comorbidity Index (CCI) scores were calculated with claims from one year prior to cancer diagnosis, excluding cancer and CVD outcomes because everyone in the study had cancer and CVD outcomes were accounted for individually. Prevalence for obesity and tobacco use disorder before cancer diagnosis were also identified from ICD codes with the Chronic Conditions Data Warehouse (27 CCW Chronic Conditions Algorithms) [27]. Medicare claims were also used to supplement SEER data for receiving chemotherapy and radiation therapy, which is often underestimated in SEER data alone [28].

### Statistical analysis

Chi-square tests were used to compare demographic and clinical covariates for NHW, East Asian, Southeast Asian, South Asian, and NHPI CRC survivors. To examine how the risk of CVD may differ over follow-up periods, we modelled three different periods after cancer diagnosis: > 1 year, > 1–5 years, and > 5 years. For each period, prevalent cases of CVD were removed for each respective CVD outcome. For example, if a patient had a prevalent claim for heart failure but not for ischemic heart disease or stroke/transient ischemic attacks, they would be removed for only the hazard ratio calculations for heart failure and composite CVD. Patients who died during follow-up or did not develop the CVD outcome of interest by the end of follow-up were censored. Cox proportional hazards (PH) models were used to calculate hazard ratios (HR) and 95% confidence intervals (CI) comparing the risk for composite CVD and each CVD event among CRC survivors of each regional subgroup versus NHW CRC survivors. While the NHW group is not required to demonstrate heterogeneity of CVD within the ANHPI regional subgroups, we decided to include this group to give context to how each ANHPI race and ethnicity exhibits different risk of CVD compared to the general US population. The models were stratified on matched pairs and adjusted for potential confounders, including SEER cancer registry, census tract-level education and household income, and rural residence. A sensitivity analysis was conducted adjusting for baseline CCI score, obesity before cancer diagnosis, and tobacco use disorder before cancer diagnosis in addition to the potential confounders. Additionally, Cox PH models were performed with East Asians, the largest ANHPI regional subgroup, as the reference to investigate heterogeneity among the ANHPI subgroups and differences with NHWs. Our study examines multiple CVD outcomes as individual endpoints, with common risk factors and etiologies. While these hypothesis tests may be expected to be highly correlated, we prespecified all CVD outcomes with distinct diagnosis codes and perform our analyses separately for each outcome. Additionally, we report all our findings and focus on inferences based on the magnitude and direction of the point estimate, the 95% confidence interval, and consistency of the associations that we observe across different follow-up periods. For our post-hoc analysis, we performed subgroup analyses by sex and by individual race and ethnicity groups to investigate differences in CVD risk among these subgroups. For the individual race and ethnicity subgroup analysis, we used Japanese CRC survivors as our reference group since they are the largest individual race and ethnicity group among ANHPI CRC patients. Additionally, we conducted a risk factor analysis to further identify differences in demographic and clinical factors that may affect ANHPI and NHW CRC survivors and their risk of heart failure and ischemic heart disease.

To conduct a complete-cases analysis, patients with missing information for age at cancer diagnosis, sex, education, household income, SEER registry, rural status, baseline Charlson comorbidity index, obesity before cancer diagnosis, tobacco use disorder before cancer diagnosis, month of diagnosis, and cancer stage were excluded from the study. The PH assumption was assessed for each Cox PH model by including an interaction term between the predictors and time in the model. For models that violated the PH assumption, estimates were calculated using flexible parametric models with restricted cubic splines in Stata/SE 17 (StataCorp, 2021) and compared to estimates from the Cox PH models [29]. All other analyses were conducted SAS 9.4 (SAS Institute, Inc, NC).

## Results

A total of 85,357 CRC survivors were identified from the SEER-Medicare dataset using our inclusion and exclusion criteria. After excluding those with missing demographic characteristics, census-level education (n = 86), census-level household income (n = 89), and rural status(n = 6), 85,268 CRC survivors were included in our cohort prior to matching. After matching by sex, age at cancer diagnosis, and year of cancer diagnosis, our analytical cohort included 17,084 ANHPI and NHW CRC survivors: 12,813 NHW CRC survivors, 2,772 East Asian CRC survivors, 1,108 Southeast Asian CRC survivors, 175 CRC survivors, and 216 NHPI CRC survivors (Fig. 1). The mean follow-up time of our analytical cohort was 6.1 years, with a range between 1 and 20 years.

Baseline demographic characteristics for our cohort are shown in Table 1. Compared to NHW CRC survivors, East Asian CRC survivors had the highest age at cancer diagnosis, followed by NHWs, Southeast Asian, South Asian, and NHPI CRC survivors. Southeast Asians had the lowest census-level education among all groups, while NHW CRC survivors had the lowest census-level household income. All ANHPI subgroups resided primarily in the West SEER registry region. The majority of East Asian, Southeast Asian, and South Asian CRC survivors resided in urban regions, while a lower proportion of NHW and NHPI resided urban regions. Southeast Asians had the highest proportion of participants with higher baseline CCI scores, with NHW and NHPI having the lowest baseline comorbidities. However, NHW and NHPI CRC survivors had the highest proportion of obesity and tobacco use disorder prior to cancer diagnosis.

Clinical characteristics of the study population are shown in Table 2. NHW and East Asian CRC survivors had a greater proportion of proximal colon cancer while South Asian CRC survivors had a greater proportion of distal colon cancer. Southeast Asian and NHPI CRC survivors had similar proportion across the three main colorectal cancer subsites. For cancer stage at diagnosis, while all ANHPI subgroup participants were mainly diagnosed in the regional stage, South Asian and NHPI CRC survivors had a higher proportion diagnosed at the distant stage. NHW CRC survivors had the highest proportion diagnosed at the localized stage while East Asian and Southeast Asian had similar distributions with more localized and regional stage at cancer diagnosis compared to the other ANHPI subgroups. For surgery, the majority of participants received surgery, while South Asian CRC survivors had the lowest proportion receiving surgery among all groups. NHW CRC survivors had the lowest proportion of those who received chemotherapy. Overall, NHW and ANHPI CRC survivors had similar proportions of receiving radiation therapy and chemotherapy. However, East Asian CRC survivors had a slightly lower proportion who received radiation therapy and Southeast Asian CRC survivors had a higher proportion.

**Table 2:** Clinical characteristics of NHW, Asian, and NHPI colorectal cancer survivors from SEER-Medicare data, by regional subgroup (*n* = 17,084)

	**NHW (*n* = 12,813)**	**East Asian (*n* = 2772)**	**Southeast Asian (*n* = 1108)**	**South Asian (*n* = 175)**	**NHPI (*n* = 216)**
Subsite, organ					
Proximal Colon	6695 (52.3)	1237 (44.6)	390 (35.2)	49 (28.0)	79 (36.6)
Distal Colon	3040 (23.7)	872 (31.5)	371 (33.5)	72 (41.1)	67 (31.0)
Rectum	3078 (24.0)	663 (23.9)	347 (31.3)	54 (30.9)	70 (32.4)
Subsite					
Proximal Colon					
Cecum	2596 (20.3)	397 (14.3)	123 (11.1)	22 (12.6)	30 (13.9)
Ascending colon	2251 (17.6)	445 (16.1)	137 (12.4)	14 (8.0)	22 (10.2)
Hepatic Flexure	554 (4.3)	122 (4.4)	37 (3.3)	**	**
Transverse colon	1012 (7.9)	213 (7.7)	64 (5.8)	**	15 (6.9)
Splenic Flexure	282 (2.2)	60 (2.2)	29 (2.6)	**	**
Distal Colon					
Descending Colon	493 (3.8)	131 (4.7)	61 (5.5)	**	> 5 (> 2.9)^a^
Sigmoid Colon	2313 (18.1)	714 (25.8)	296 (26.7)	47 (26.9)	51 (23.6)
Large Intestine, NOS	234 (1.8)	27 (1.0)	14 (1.3)	**	< 11 (< 6.3)^a^
Rectum					
Rectosigmoid Junction	920 (7.2)	216 (7.8)	115 (10.4)	18 (10.3)	20 (9.3)
Rectum	2158 (16.8)	447 (16.1)	232 (20.9)	36 (20.6)	50 (23.1)
Cancer Stage					
Localized	6009 (46.9)	1164 (42.0)	429 (38.7)	68 (38.9)	95 (44.0)
Regional	5531 (43.2)	1318 (47.5)	552 (49.8)	80 (45.7)	89 (41.2)
Distant	1273 (9.9)	290 (10.5)	127 (11.5)	27 (15.4)	32 (14.8)
AJCC Stage					
I	3383 (26.4)	681 (24.6)	239 (21.6)	29 (16.6)	56 (25.9)
II	3948 (30.8)	790 (28.5)	284 (25.6)	46 (26.3)	52 (24.1)
III	2961 (23.1)	787 (28.4)	323 (29.2)	44 (25.1)	55 (25.5)
IV	1014 (7.9)	244 (8.8)	94 (8.5)	12 (6.9)	25 (11.6)
Unknown/Missing	1507 (11.8)	270 (9.7)	168 (15.2)	44 (25.1)	28 (13.0)
Surgery					
No	686 (5.4)	> 127 (> 4.6)^a^	> 71 (> 6.4)^a^	> 15 (> 8.6)^a^	> 8 (> 3.7)^a^
Yes	12,111 (94.5)	2634 (95.0)	1026 (92.6)	149 (85.1)	197 (91.2)
Unknown	16 (0.1)	< 11 (< 6.3)^a^	< 11 (< 6.3)^a^	< 11 (< 6.3)^a^	< 11 (< 6.3)^a^
Radiation Therapy					
No	11,106 (86.7)	2434 (87.8)	929 (83.8)	149 (85.1)	185 (85.6)
Yes	1707 (13.3)	338 (12.2)	179 (16.2)	26 (14.9)	31 (14.4)
Chemotherapy					
No	7851 (61.3)	1637 (59.1)	613 (55.3)	93 (53.1)	125 (57.9)
Yes	4962 (38.7)	1135 (40.9)	495 (44.7)	82 (46.9)	91 (42.1)

*NHW* Non-Hispanic White, *NHPI* Native Hawaiian and Pacific Islander

^a^**Cell value coarsened due to CMS cell size suppression policy

At cancer diagnosis, South Asian CRC survivors had the highest prevalence of composite CVD, heart failure, and ischemic heart disease compared to NHW and other ANHPI regional subgroup CRC survivors (Supplemental Table 2). Compared to NHW CRC survivors, East Asian, Southeast Asian, and NHPI CRC survivors had a lower baseline prevalence of composite CVD, heart failure, and ischemic heart disease. Compared to East Asian CRC survivors, NHPI CRC survivors had a higher prevalence of heart failure. The prevalence of stroke/transient ischemic attack was similar across all groups. Throughout the whole study period, there were about 2,219 incident cases of heart failure, 1,507 incident cases of ischemic heart disease, and 1,310 incident cases of stroke/transient ischemic attack (Supplemental Table 3). The cumulative risk for our full study period was 18.3% for heart failure, 17.5% for ischemic heart disease, and 9.2% for stroke/transient ischemic attack. NHW colorectal cancer survivors had the highest cumulative risk for heart failure and ischemic heart disease compared to the ANHPI regional subgroups. Among ANHPI subgroups, NHPI CRC survivors had the highest cumulative risk of heart failure while South Asians had the lowest. NHPI CRC survivors had the lowest cumulative risk of ischemic heart disease.

**Table 3:** Risk of cardiovascular disease incidence among ANHPI and NHW colorectal cancer survivors diagnosed 2000 to 2017 from SEER-Medicare data, stratified by follow-up period and regional subgroup (reference = NHW)

	**NHW**	**East Asian**	**Southeast Asian**	**South Asian**	**NHPI**
	HR (95% CI)	HR (95% CI)	HR (95% CI)	HR (95% CI)	HR (95% CI)
All					
Composite CVD	Reference	0.74(0.64,0.86)^a^	0.92(0.75,1.12)^a^	1.06(0.66,1.69)^a^	0.84(0.56,1.25)^a^
Heart Failure	Reference	0.64(0.52,0.78)	0.88(0.68,1.14)	0.83(0.42,1.64)	0.93(0.56,1.54)
Ischemic Heart Disease	Reference	0.68(0.58,0.81)^a^	0.99(0.80,1.23)^a^	1.24(0.74, 2.08)^a^	0.75(0.47,1.22)^a^
Stroke/Transient Ischemic Attack	Reference	1.00(0.79,1.28)	1.06(0.75,1.51)	1.06(0.47,2.39)	0.88(0.46,1.69)
> 1–5 years					
Composite CVD	Reference	0.80(0.60,1.07)	1.22(0.86,1.74)	1.41(0.63,3.15)	0.94(0.45,1.94)
Heart Failure	Reference	0.68(0.54,0.86)^a^	1.07(0.82,1.41)^a^	0.94(0.48,1.82)^a^	0.96(0.54,1.71)^a^
Ischemic Heart Disease	Reference	0.67(0.48,0.93)	1.42(0.98,2.07)	2.17(0.88,5.37)	0.75(0.32,1.71)
Stroke/Transient Ischemic Attack	Reference	1.17(0.83,1.65)	1.27(0.79,2.05)	1.78(0.71,4.45)	1.13(0.46,2.79)
> 5 years					
Composite CVD	Reference	0.58(0.39,0.87)	0.56(0.29,1.06)	0.20(0.02,1.78)	0.35(0.12,1.01)
Heart Failure	Reference	0.50(0.35,0.71)	0.64(0.40,1.01)	0.76(0.19,3.08)	0.67(0.27,1.65)
Ischemic Heart Disease	Reference	0.46(0.29,0.72)	0.37(0.18,0.75)	1.16(0.16,8.24)	0.44(0.15,1.28)
Stroke/Transient Ischemic Attack	Reference	0.77(0.49,1.20)	0.76(0.39,1.46)	**	0.64(0.21,1.97)

*ANHPI* Asian, Native Hawaiian, and Pacific Islander, *CVD* Cardiovascular disease, *NHW* Non-Hispanic White, *NHPI* Native Hawaiian and Pacific Islander, *95% CI* 95% confidence interval

Models adjusted for SEER registry, income, college education, and rural status

^a^Proportional hazard assumption not met; flexible spline model used

^**^Cell value coarsened due to CMS cell size suppression policy

For composite CVD, heart failure, and ischemic heart disease, East Asians had a lower risk compared to their NHW counterparts for the full study follow-up (Table 3). For the > 1–5-year period, East Asian CRC survivors had a lower risk of heart failure and ischemic heart disease compared to their NHW counterparts. For this same period, the magnitude of the point estimate for South Asian CRC survivors for ischemic heart disease suggested an elevated risk but was also compatible with a null association. For > 5 years of follow-up, East Asian and Southeast Asian CRC survivors had an observed lower risk of composite CVD, heart failure, and ischemic heart disease compared to the NHW counterparts. There were no associations observed for stroke/transient ischemic attack. For all follow-up periods, NHPI CRC survivors had similar risk of all CVD outcomes compared to their NHW counterparts. In our sensitivity analysis that additionally adjusting for baseline CCI, obesity before cancer diagnosis, and tobacco use disorder before cancer diagnosis, the associations were similar to the models that did not control these baseline conditions (Supplemental Table 4).

**Table 4:** Risk of cardiovascular disease incidence among ANHPI and NHW colorectal cancer survivors diagnosed 2000 to 2017 from SEER-Medicare data, stratified by follow-up period and regional subgroup (reference = East Asian)

	**NHW**	**East Asian**	**Southeast Asian**	**South Asian**	**NHPI**
	HR (95% CI)	HR (95% CI)	HR (95% CI)	HR (95% CI)	HR (95% CI)
All					
Composite CVD	1.34(1.16,1.56)^a^	Reference	1.23(1.00, 1.15)^a^	1.42(0.88,2.30)^a^	1.12(0.76,1.67)^a^
Heart Failure	1.57(1.28,1.91)	Reference	1.38(1.04,1.84)	1.30(0.65,2.62)	1.45(0.87,2.42)
Ischemic Heart Disease	1.46(1.23,1.74)^a^	Reference	1.45(1.15,1.82)^a^	1.81(1.07,3.09)^a^	1.10(0.69,1.77)^a^
Stroke/Transient Ischemic Attack	1.00(0.78,1.27)	Reference	1.06(0.73,1.55)	1.06(0.46,2.44)	0.88(0.46,1.68)
> 1–5 years					
Composite CVD	1.25(0.94,1.66)	Reference	1.52(1.03,2.25)	1.76(0.76,4.06)	1.17(0.56,2.43)
Heart Failure	1.47(1.17,1.84)^a^	Reference	1.58(1.17,2.12)^a^	1.37(0.69,2.72)^a^	1.41(0.79,2.51)^a^
Ischemic Heart Disease	1.50(1.08,2.08)	Reference	2.13(1.39,3.28)	3.26(1.25,8.45)	1.12(0.48,2.58)
Stroke/Transient Ischemic Attack	0.86(0.61,1.20)	Reference	1.09(0.65,1.82)	1.52(0.58,3.96)	0.97(0.40,2.36)
> 5 years					
Composite CVD	1.59(1.17,2.15)	Reference	0.86(0.52,1.44)	0.46(0.09,2.42)	0.94(0.43,2.04)
Heart Failure	1.76(1.32,2.35)	Reference	1.17(0.77,1.79)	1.32(0.38,4.55)	1.63(0.75,3.53)
Ischemic Heart Disease	1.91(1.33,2.75)	Reference	0.98(0.55,1.74)	2.43(0.58,10.14)	1.19(0.51,2.78)
Stroke/Transient Ischemic Attack	1.15(0.78,1.70)	Reference	0.81(0.43,1.54)	0.48(0.04,5.21)	0.64(0.22,1.84)

Models adjusted for SEER registry, income, college education, and rural status

*ANHPI* Asian, Native Hawaiian, and Pacific Islander, *CVD* Cardiovascular disease, *NHW* Non-Hispanic White, *NHPI* Native Hawaiian and Pacific Islander, *95% CI* 95% confidence interval

^a^Proportional hazard assumption not met; flexible spline model used

When compared to East Asian CRC survivors, we observed a greater risk of CVD outcomes among the other ANHPI regional subgroups (Table 4). For all follow-up time, NHW and Southeast Asian CRC survivors had a higher risk of composite CVD, heart failure, and ischemic heart disease. South Asian CRC survivors had a higher risk of ischemic heart disease. Similar associations were observed for NHW, Southeast Asian, and South Asian CRC survivors for the > 1–5-year follow-up period. For the > 5 years, NHW CRC survivors had a higher risk of composite CVD, heart failure, and ischemic heart disease. Similarly to the comparison with NHW CRC survivors, the magnitude of the point estimate for ischemic heart disease among South Asian CRC survivors suggested an elevated risk but was also compatibly with a null association. The subgroup analysis by individual race and ethnicity further discerned differences among ANHPI groups (Supplemental Table 5). With NHW CRC survivors as the reference group, Japanese CRC survivors had a lower risk of composite CVD, heart failure, and ischemic heart disease, while their Chinese counterparts had a lower risk of composite CVD and heart failure. When compared to Japanese CRC survivors, NHW and Filipino CRC survivors had elevated risk of composite CVD, heart failure, and ischemic heart disease. Additionally, Asian Indian and Pakistani CRC survivors had an elevated risk of ischemic heart disease compared to Japanese CRC survivors.

**Table 5:** Risk factors for cardiovascular disease incidence in NHW and ANHPI colorectal cancer survivors for > 5 years after cancer diagnosis

	**Heart Failure**		**Ischemic Heart Disease**	
	NHW (*n* = 4799)	ANHPI (*n* = 1730)	NHW (*n* = 3786)	ANHPI (*n* = 1364)
	HR (95% CI)	HR (95% CI)	HR (95% CI)	HR (95% CI)
Sex				
Female	Reference	Reference	Reference	Reference
Male	1.13(1.00,1.29)^#^	1.28(1.00,1.64)	1.37(1.18,1.60)	1.19(0.89,1.59)
Age at Cancer Diagnosis^a^				
66 to 70	Reference	Reference	Reference	Reference
71 to 75	1.21(1.01,1.46)	1.43(0.98,2.10)	1.14(0.93,1.39)	1.13(0.75,1.69)
76 to 80	1.98(1.65,2.38)	2.27(1.55,3.21)	1.38(1.12,1.70)	1.33(0.87,2.03)
81 to 100	2.60(2.12,3.19)	2.63(1.74,3.99)	1.41(1.10,1.81)	1.84(1.15,2.96)
Education^f^				
< = 40% had some college education	Reference	Reference	Reference	Reference
40%- < = 60% had some college education	0.95(0.77,1.17)	1.35(0.91,2.00)	0.74(0.58,0.94)	0.71(0.46,1.11)
60%- < = 80% had some college education	0.95(0.75,1.22)	1.12(0.76,1.91)	0.73(0.56,0.97)	0.84(0.50,1.39)
> 80% had some college education	0.86(0.62,1.19)	1.72(0.96,3.08)	0.60(0.41,0.87)	0.94(0.48,1.85)
Household Income^g^				
< $50,000	Reference	Reference	Reference	Reference
$50,000 to < $60,000	0.98(0.80,1.21)^#^	0.94(0.66,1.36)	1.10(0.87,1.39)	0.73(0.46,1.16)
$60,000 to < $70,000	1.07(0.84,1.36)^#^	0.59(0.37,0.95)	1.14(0.86,1.50)	0.65(0.38,1.12)
$70,000 +	1.14(0.90,1.44)^#^	0.67(0.44,1.02)	1.10(0.84,1.45)	0.75(0.46,1.22)
Rural Status				
Urban	Reference	Reference	Reference	Reference
Rural	0.89(0.75,1.05)	1.51(0.96,2.39)	0.96(0.80,1.16)	1.54(0.92,2.57)
Baseline CCI^2,b^				
0	Reference	Reference	Reference	Reference
1	1.60(1.38,1.86)	1.90(1.44,2.51)	1.62(1.35,1.93)	1.32(0.93,1.87)
2 +	1.91(1.52,2.40)	2.04(1.38,3.02)	2.27(1.74,2.94)	2.37(1.53,3.69)
Obesity before cancer diagnosis^c^				
No	Reference	Reference	Reference	Reference
Yes	1.39(1.05,1.86)	2.47(1.08,5.65)	1.59(1.14,2.21)	0.83(0.11,5.98)
Tobacco use disorder before cancer diagnosis^c^				
No	Reference	Reference	Reference	Reference
Yes	2.28(1.74,2.99)	1.43(0.58,3.48)	1.66(1.12,2.46)	0.51(0.07,3.68)
Cancer Stage^d^				
Localized	Reference	Reference	Reference	Reference
Regional	1.04(0.91,1.18)	0.91(0.71,1.18)	0.95(0.81,1.10)	1.08(0.79,1.46)
Distant	0.39(0.18,0.82)	0.42(0.10,1.73)	0.89(0.51,1.56)	1.39(0.56,3.48)
Surgery^e^				
No	Reference	Reference	Reference	Reference
Yes	1.39(0.62,3.12)	0.30(0.13,0.69)	0.80(0.41,1.56)	0.86(0.25,2.87)
Unknown				
Radiation Therapy^e^				
No	Reference	Reference	Reference	Reference
Yes	0.86(0.69,1.08)	1.03(0.66,1.61)	1.16(0.92,1.45)	0.96(0.60,1.54)
Chemotherapy^e^				
No/unknown	Reference	Reference	Reference	Reference
Yes	1.02(0.87,1.20)	0.98(0.70,1.37)	1.07(0.89,1.29)	0.91(0.62,1.35)

*NHW* Non-Hispanic White, *ANHPI* Asian, Native Hawaiian, and Pacific Islander, *HR* Hazard ratio, *AJCC* American Joint Committee on Cancer; CCI, Charlson Comorbidity Index; 95% CI, 95% confidence interval

^1^Census-level data

^2^CCI excluded cardiovascular conditions

^a^Adjusted for sex, diagnosis year, census-level college education, census-level household income, SEER registry, rural status, modified CCI, obesity before cancer diagnosis, tobacco use disorder before cancer diagnosis, and cancer stage

^b^Adjusted for sex, diagnosis age, diagnosis year, census-level college education, cenus-level household income, SEER registry, rural status, obesity before cancer diagnosis, and tobacco use before cancer diagnosis

^c^Adjusted for sex, census-level college education, cenus-level household income, SEER registry, and rural status

^d^Adjusted for sex, diagnosis age, diagnosis year census-level college education, cenus-level household income, SEER registry, baseline CCI, obesity before cancer diagnosis, and tobacco use disorder before cancer diagnosis

^e^Adjusted for sex, diagnosis age, diagnosis year, census-level college education, census-level household income, SEER registry, rural status, baseline CCI, obesity before cancer diagnosis, tobacco use before cancer diagnosis, and cancer stage

^f^Adjusted for census-level household income, SEER registry, and rural status

^g^Adjusted for census-level college education, SEER registry, and rural status

^#^Proportional hazard assumption not met; flexible spline model used

For the risk factor analysis, we observed differences in risk and protective factors for heart failure and ischemic heart disease for NHW and ANHPI CRC survivors for > 5 years of follow-up (Table 5). For both heart failure ischemic heart disease, older age at cancer diagnosis and higher baseline CCI scores were associated with higher risk among ANHPI and NHW CRC survivors. For heart failure, being male and obesity before cancer diagnosis were associated with higher risk whereas higher income and having surgery were protective among ANHPI CRC survivors. Among NHW CRC survivors, obesity and tobacco use disorder before cancer diagnosis were associated with an increased risk of heart failure while having distant stage CRC was protective. Being male, having obesity before cancer diagnosis, and having a tobacco use disorder before cancer diagnosis were associated with an increased risk of ischemic heart disease among NHW CRC survivors. However, higher education levels were protective of ischemic heart disease among NHW CRC survivors.

When stratified by sex, male NHW CRC survivors had a higher risk of composite CVD, heart failure, and ischemic heart disease compared to their male East Asian counterparts (Table 6). Male Southeast Asian CRC survivors had a higher risk of composite CVD and ischemic heart disease, and male Southeast Asian CRC survivors had a higher risk of ischemic heart disease compared to their East Asian counterparts. The associations among the NHPI group were suggestive of elevated risk, but the wide confidence intervals were also compatible with null associations. For females, NHW CRC survivors had an increased risk of heart failure compared to female East Asian CRC survivors.

**Table 6:** Risk of cardiovascular disease incidence among ANHPI colorectal cancer survivors and NHW colorectal cancer survivors for > 1 year after cancer diagnosis, stratified by sex and regional subgroup

Males	NHW			East Asian			Southeast Asian			South Asian			NHPI		
	N	Cases	HR (95% CI)	N	Cases	HR (95% CI)	N	Cases	HR (95% CI)	N	Cases	HR (95% CI)	N	Cases	HR (95% CI)
Composite CVD	1868	562	1.90(1.32,2.73)	659	162	Reference	282	71	1.81(1.14,2.87)	43	**	2.13(0.70,6.49)	63	12	1.18(0.51,2.76)
Heart Failure	4142	836	1.46(1.09,1.97)	1110	173	Reference	457	72	1.44(0.97,2.15)	77	**	1.62(0.67,3.91)	93	16	1.57(0.74,3.34)
Ischemic Heart Disease	2398	508	2.18(1.46,3.24)	794	120	Reference	343	62	2.12(1.29,3.49)	50	**	4.23(1.13,15.77)	71	**	1.23(0.50,3.05)
Stroke/Transient Ischemic Attack	5070	443	0.88(0.61,1.27)	1174	113	Reference	514	44	0.99(0.58,1.67)	85	**	0.80(0.19,3.37)	101	**	1.26(0.46,3.48)
Females	NHW			East Asian			Southeast Asian			South Asian			NHPI		
	N	Cases	HR (95% CI)	N	Cases	HR (95% CI)	N	Cases	HR (95% CI)	N	Cases	HR (95% CI)	N	Cases	HR (95% CI)
Composite CVD	2588	728	1.23(0.93,1.61)	874	223	Reference	313	60	1.17(0.76,1.82)	47	**	0.81(0.31,2.13)	66	15	0.93(0.45,1.94)
Heart Failure	4431	857	1.62(1.23,2.14)	1246	180	Reference	438	52	1.33(0.87,2.03)	64	**	1.00(0.30,3.31)	89	17	1.31(0.65,2.68)
Ischemic Heart Disease	3422	579	1.22(0.90,1.67)	1051	153	Reference	370	51	1.39(0.85,2.26)	55	**	1.58(0.54,4.66)	81	**	0.93(0.37,2.33)
Stroke/Transient Ischemic Attack	5399	523	1.09(0.78,1.52)	1343	137	Reference	498	33	1.12(0.63,1.97)	73	**	1.27(0.44,3.67)	92	**	0.67(0.28,1.61)

Models adjusted for SEER registry, income, college education, and rural status

*ANHPI* Asian, Native Hawaiian, and Pacific Islander, *CVD* Cardiovascular disease, *NHW* Non-Hispanic White, *NHPI* Native Hawaiian and Pacific Islander, *95% CI* 95% confidence interval

^a^Proportional hazard assumption not met; flexible spline model used

^**^Cell values suppressed due to the Centers for Medicare & Medicaid Services (CMS) cell size suppression policy

## Discussion

In this study, we observed CVD disparities among older Asian, Native Hawaiian, and Pacific Islander CRC survivors by regional subgroups. Our results showed that when compared to older NHW CRC survivors, there was observed differences in risk among ANHPI subgroups, specifically for CVD among East Asian and Southeast Asians. However, when compared to East Asians, there were increased risk of composite CVD, heart failure, and ischemic heart disease among Southeast Asian and South Asian CRC survivors across different follow-up periods. More specifically, South Asian CRC survivors had a higher risk of ischemic heart disease.

From our analysis, the risk of composite CVD, heart failure, and ischemic heart disease was lower for older East Asian CRC survivors compared to older NHW CRC survivors. A potential explanation for the lower risk among East Asian CRC survivors may be their lower prevalence of risk factors associated with CVD. In our cohort, East Asian CRC survivors had higher levels of education and household income, while having lower levels of obesity and tobacco use disorder before cancer diagnosis. NHW CRC survivors had a higher proportion residing in rural areas, lower census-level college education, and household income compared to their ANHPI counterparts. Financial burden, lack of access and transportation to treatment, and scarcity of physicians and services have been previously observed as healthcare barriers among rural populations [30]. In this study, NHPI CRC survivors also had proportions of rural residence and lower levels of college education similar to NHW CRC survivors. NHPI CRC survivors also had a higher prevalence of CVD risk factors such as obesity, tobacco use disorder, and low socioeconomic status. They also had slightly lower proportions of receiving surgery, chemotherapy, and radiation therapy. These disparities may explain some of the similarities in risks of CVD among older NHPI and NHW CRC survivors and the heterogeneity among the ANHPI CRC regional subgroups.

Older South Asian CRC survivors had a higher risk of ischemic heart disease compared to older East Asian CRC survivors. Other studies have reported that South Asians in the US have the highest rates of diabetes and insulin resistance, which increases their susceptibility to atherosclerotic cardiovascular disease [31]. South Asians may also be at a higher risk of CVD due to their genetic predisposition to polymorphisms in the *APOA1*gene that results in dyslipidemia [32]. We also observed that Southeast Asian CRC survivors, specifically Filipinos, had a higher risk of incident heart failure and ischemic heart disease compared to East Asian CRC survivors. In a study examining heart failure data from the Oracle EHR Real-World Data database, Southeast Asians and South Asians had higher age- and sex-standardized incidence of heart failure compared to East Asians, with the incidence difference between Southeast Asians and East Asians being greater than the disparities between Black and White patients [33]. The differences in heart failure within the Asian subgroups may be due disparities in insurance coverage, financial hardships, immigration history, and assimilation to Western culture. Among Southeast Asians, Filipinos in the US have been previously reported to have the highest burden of ischemic heart disease among Asians [34]. Our results are consistent with these prior studies, as this group had a higher risk of ischemic heart disease. In a similar study examining CVD disparities among disaggregated ANHPI race and ethnicity groups among breast cancer survivors, Filipino, Asian Indian and Pakistani, and Native Hawaiian breast cancer survivors had a higher risk of heart failure and ischemic heart disease, which was consistent to our results among Southeast Asian and South Asian CRC survivors [25]. In another SEER-Medicare study examining ANHPI regional subgroups and lung cancer, Pacific Islanders, South Asian, and Southeast Asian lung cancer survivors were found to have higher risk of heart failure compared to their Chinese counterparts [26]. This indicates a higher risk of CVD outcomes in these ANHPI subgroups across CRC, breast cancer, and lung cancer.

The risk of stroke/transient ischemic attack did not differ among NHW and ANHPI regional subgroups. However, prior studies in general populations have reported stroke rates to be nearly four times greater among NHPIs compared to Asians [22, 35]. Transient ischemic attack cases may be underreported in claims data, as the symptoms are acute and often resolve before clinical assessment [36, 37]. This may result in a smaller sample size of patients with this outcome of interest, leading to an underestimate of the risk of stroke/transient ischemic attack and bias towards the null.

We observed heterogeneity for CVD risk factors among ANHPI CRC survivor race and ethnicity groups. Older age at cancer diagnosis, higher CCI score, having obesity before cancer diagnosis, and having tobacco use disorder before cancer diagnosis were identified as risk factors for heart failure and ischemic heart disease. Other risk factors that may influence CVD risk include low socioeconomic status, diabetes, tobacco use, and alcohol use [38–40]. In this risk factor analysis, we grouped Asians and NHPI together due to insufficient sample size when stratifying by risk factors. Although often aggregated together, ANHPI CRC survivors differ in these risk factors, resulting in differences towards their susceptibility to CVD. Compared to Asian, NHPIs had higher prevalence of tobacco use, obesity and diabetes [41–43]. With the aggregated Asian group, Chinese CRC survivors had the lowest prevalence of diabetes and obesity, whereas Indians and Filipinos had the highest [44, 45]. Another observational study observed differences in social determinants of health that varied among Asian subgroups and may increase CVD risk, including physical activity, neighborhood cohesion, health literacy, English proficiency, and food security [46]. In immigrant studies, Chinese and Japanese immigrants were reported to have lower rates of heart disease due to lower prevalence of obesity, smoking, and alcohol consumption [19, 47]. Among Asians, previous studies have also reported that Filipinos have the highest prevalence of type 2 diabetes, hypertension, and obesity [48]. A study from the National Health Interview Survey reported that US-born Filipino adults had higher likelihood of comorbidities of both coronary artery disease and atherosclerotic cardiovascular disease [49]. The higher prevalence of these conditions and the CVD risk factors may be a potential pathway for the higher incidence of heart failure and ischemic heart disease among older Filipino CRC survivors in our study.

A strength of our study included the use of the SEER-Medicare dataset to assess CVD risk in CRC survivors by ANHPI regional subgroups, which has not been done before according to our knowledge. Previous studies tended to group ANHPI race and ethnicities, even with the known differences in cultural, genetic, and health-related factors. We observed heterogeneity in CVD risk among Asians and NHPIs, as well as within Asian race and ethnicity groups. The SEER linkage with Medicare claims allowed for adjustment for known demographic and clinical risk factors for both CRC and CVD. We were also able to examine long follow-up periods, which minimized the likelihood of surveillance bias which is a concern for cancer patients after their diagnosis. By using Medicare claims data to assess CVD incidence, we minimized recall errors associated with self-reporting.

A limitation of this study was the lack of data on behavioral risk factors [50]. Proxies for behavioral risk factors were derived from Medicare claim ICD diagnostic codes for obesity and tobacco use disorders, which tend to have low sensitivity. Another potential limitation is missing diagnoses for CVD events that may occur prior to Medicare eligibility. To account for unknown conditions prior to Medicare enrollment, our study excludes patients with less than one year of follow-up. This may reduce the number of cases, specifically those associated with cardiotoxicity from surgical, chemotherapy, and radiation therapy within the 1 st year after cancer diagnosis [51]. However, this exclusion allows us to conduct time-to-event analysis more accurately for the CVD outcomes. In addition, our exclusion criteria may limit the generalizability of our results for colorectal cancer survivors without continuous Medicare Part A/B coverage and those with HMO enrollment. While this may restrict generalizability, this restriction ensured that we had complete claims data for our cohort, allowing us to ascertain all incident CVD events. While there is a possibility of competing risk due to the potential of death from cancer prior to incident CVD, we do not expect substantial difference in death among the ANHPI racial/ethnicity groups and therefore the relative risks are unlikely biased by competing risk. Additionally, our results do not apply to CRC survivors diagnosed at < 66 years of age. The increasing incidence of early-onset CRC warrants further investigation of CRC-related CVD events among younger CRC survivor populations. Another potential limitation is the lack of information about people with multiple racial and/or ethnic identities. The race and ethnicity variable in the SEER-Medicare dataset does not provide information among individuals of mixed race, so this may lead to non-differential misclassification and bias towards the null. However, the SEER database prioritizes non-White races, with highest priority to Native Hawaiians followed by other race and ethnicities. While our study was unable to factor in multiple races and ethnicities, we were able to examine those who primarily identified themselves as ANHPI. Furthermore, even with the large sample size, we still had sparse data for individual ethnicity groups, especially among South Asian and NHPI CRC survivors. While the direction of the point estimates is suggestive of higher risk of CVD outcomes compared to their East Asian counterparts, the small sample size results in imprecise intervals that are also compatible with null associations. However, our study was able to identify heterogeneity in CVD risk for ANHPIs and regional subgroups for colorectal cancer survivors, which has not been done before. More studies are needed to better understand CVD risk among NHPI and underrepresented Asian CRC survivor groups.

## Conclusion

ANHPI CRC survivors are often understudied for long-term health outcomes as they are considered healthier relative to other race and ethnicity groups. They have the lowest rates of incident CRC and CVD events of all race and ethnicity groups, but these estimates overlook the heterogeneity within the ANHPI population. Our findings support the presence of health disparities in CVD risk factors and incidence among the disaggregated ANHPI race and ethnicities, with higher risk among older South Asian and Southeast Asian CRC survivors. The heterogeneity in factors such as culture, diet, and access to healthcare need more attention for ANHPI CRC survivors. Further studies are needed to explore these factors that may contribute to the greater burden in these high CVD risk populations with the ANHPI grouping.

## Supplementary Information

Supplementary Material 1. Supplemental Table 1. ICD Codes for cardiovascular disease outcomes from the CCW Chronic Conditions Algorithm. Supplemental Table 2. Prevalence of cardiovascular disease at cancer diagnosis among colorectal cancer survivors diagnosed 2000 to 2017 from SEER-Medicare data (n=17804). Supplemental Table 3. Sample size, incident cases, and cumulative risk for risk of cardiovascular disease incidence among ANHPI and NHW colorectal cancer survivors diagnosed 2000 to 2017 from SEER-Medicare data, stratified by follow-up period and regional subgroup. Supplemental Table 4. Sensitivity analysis for risk of cardiovascular disease incidence among ANHPI and NHW colorectal cancer survivors diagnosed 2000 to 2017 from SEER-Medicare data, stratified by follow-up period and regional subgroup. Supplemental Table 5. Risk of cardiovascular disease incidence among ANHPI colorectal cancer survivors and NHW colorectal cancer survivors for >1 year after cancer diagnosis, stratified by individual ANHPI race and ethnicity groups.

## Data Availability

The data that support the findings of this study are available from SEER-Medicare, but restrictions apply to the availability of these data, which were used under license for the current study, and so are not publicly available. Data are, however, available from SEER-Medicare with permission of SEER-Medicare.

## Abbreviations

ANHPI: Asian, Native Hawaiian, and Pacific Islander
CCI: Charlson Comorbidity Index
CI: Confidence Interval
CRC: Colorectal Cancer
CVD: Cardiovascular Disease
HMO: Health Maintenance Organization
HR: Hazard Ratio
ICD: International Classification of Disease
ICD-9: International Classification of Disease, Ninth Revision
ICD-10: International Classification of Disease, Tenth Revision
ICD-O-3: International Classification of Diseases for Oncology, 3rd Edition
NHPI: Native Hawaiian and Pacific Islander
NHW: Non-Hispanic White
PH: Proportional Hazards
SEER: Surveillance, Epidemiology, and End Results
