# Supplementary material for: Risk of incident cardiovascular disease events among older Asian, Native Hawaiian, and Pacific Islander colorectal cancer survivors in the United States: a cohort study
Source: Cardiooncology. 2026 Jan 12;12:23. doi: 10.1186/s40959-025-00440-4 (PMC12888620; doi:10.1186/s40959-025-00440-4)
Supplement: Supplementary file 1 — Supplementary Material 1. Supplemental Table 1. ICD Codes for cardiovascular disease outcomes from the CCW Chronic Conditions Algorithm. Supplemental Table 2. Prevalence of cardiovascular disease at cancer diagnosis among colorectal cancer survivors diagnosed 2000 to 2017 from SEER-Medicare data (n=17804). Supplemental Table 3. Sample size, incident cases, and cumulative risk for risk of cardiovascular disease incidence among ANHPI and NHW colorectal cancer survivors diagnosed 2000 to 2017 from SEER-Medicare data, stratified by follow-up period and regional subgroup. Supplemental Table 4. Sensitivity analysis for risk of cardiovascular disease incidence among ANHPI and NHW colorectal cancer survivors diagnosed 2000 to 2017 from SEER-Medicare data, stratified by follow-up period and regional subgroup. Supplemental Table 5. Risk of cardiovascular disease incidence among ANHPI colorectal cancer survivors and NHW colorectal cancer survivors for >1 year after cancer diagnosis, stratified by individual ANHPI race and ethnicity groups. [file 40959_2025_440_MOESM1_ESM.docx]

| *Supplemental Table 1. ICD Codes for cardiovascular disease outcomes from the CCW Chronic Conditions Algorithm* | | |
| --- | --- | --- |
|  | ICD-9 Codes | ICD-10 Codes |
| Acute Myocardial Infarction | 410.01, 410.11, 410.21, 410.31, 410.41, 410.51, 410.61, 410.71, 410.81, 410.91 (ONLY first or second DX on the claim) | I21.01, I21.02, I21.09, I21.11, I21.19, I21.21, I21.29, I21.3, I21.4, I21.9, I21.A1, I21.A9, I22.0, I22.1, I22.2, I22.8, I22.9 (ONLY first or second DX on the claim) |
| Heart Failure | 398.91, 402.01, 402.11, 402.91, 404.01, 404.03, 404.11, 404.13, 404.91, 404.93, 428.0, 428.1, 428.20, 428.21, 428.22, 428.23, 428.30, 428.31, 428.32, 428.33, 428.40, 428.41, 428.42, 428.43, 428.9 (any DX on the claim) | I09.81, I11.0, I13.0, I13.2, I50.1, I50.20, I50.21, I50.22, I50.23, I50.30, I50.31, I50.32, I50.33, I50.40, I50.41, I50.42, I50.43, I50.810, I50.811, I50.812, I50.813, I50.814, I50.82, I50.83, I50.84, I50.89, I50.9 (any DX on the claim) |
| Stroke/Transient Ischemic Attack | 430, 431, 433.01, 433.11, 433.21, 433.31, 433.81, 433.91, 434.00, 434.01, 434.10, 434.11, 434.90, 434.91, 435.0, 435.1, 435.3, 435.8, 435.9, 436, 997.02 (any DX on the claim) | G45.0, G45.1, G45.2, G45.8, G45.9, G46.0, G46.1, G46.2, G46.3, G46.4, G46.5, G46.6, G46.7, G46.8, G97.31, G97.32, I60.00, I60.01, I60.02, I60.10, I60.11, I60.12, I60.20, I60.21, I60.22, I60.30, I60.31, I60.32, I60.4, I60.50, I60.51, I60.52, I60.6, I60.7, I60.8, I60.9, I61.0, I61.1, I61.2, I61.3, I61.4, I61.5, I61.6, I61.8, I61.9, I63.00, I63.011, I63.012, I63.013, I63.019, I63.02, I63.031, I63.032, I63.039, I63.09, I63.10, I63.111, I63.112, I63.113, I63.119, I63.12, I63.131, I63.132, I63.133, I63.139, I63.19, I63.20, I63.211, I63.212, I63.213, I63.219, I63.22, I63.231, I63.232, I63.233, I63.239, I63.29, I63.30, I63.311, I63.312, I63.313, I63.319, I63.321, I63.322, I63.323, I63.329, I63.331, I63.332, I63.333, I63.339, I63.341, I63.342, I63.343, I63.349, I63.39, I63.40, I63.411, I63.412, I63.413, I63.419, I63.421, I63.422, I63.423, I63.429, I63.431, I63.432, I63.433, I63.439, I63.441, I63.442, I63.443, I63.449, I63.49, I63.50, I63.511, I63.512, I63.513, I63.519, I63.521, I63.522, I63.523, I63.529, I63.531, I63.532, I63.533, I63.539, I63.541, I63.542, I63.543, I63.549, I63.59, I63.6, I63.8, I63.81, I63.89, I63.9, I66.01, I66.02, I66.03, I66.09, I66.11, I66.12, I66.13, I66.19, I66.21, I66.22, I66.23, I66.29, I66.3, I66.8, I66.9, I67.841, I67.848, I67.89, I97.810, I97.811, I97.820, I97.821 (any DX on the claim) |
| Abbreviations: DX, diagnosis; ICD, International Classification of Disease | | |

| *Supplemental Table 2. Prevalence of cardiovascular disease at cancer diagnosis among colorectal cancer survivors diagnosed 2000 to 2017 from SEER-Medicare data (n=17804)* | | | | | |
| --- | --- | --- | --- | --- | --- |
|  | **NHW (n=12813)** | **East Asian (n=2772)** | **Southeast Asian (n=1108)** | **South Asian (n=175)** | **NHPI (n=216)** |
|  | N, % | N, % | N, % | N, % | N, % |
| Composite CVD | 4389 (34.3) | 760 (27.4) | 327 (29.5) | 63 (36.0) | 60 (27.8) |
| Heart Failure | 1744 (13.6) | 230 (8.3) | 130 (11.7) | 26 (14.9) | 24 (11.1) |
| Ischemic Heart Disease | 3435 (26.8) | 585 (21.1) | 256 (23.1) | 55 (31.4) | 43 (19.9) |
| Stroke/Transient Ischemic Attack | 979 (7.6) | 201 (7.3) | 80 (7.2) | ** | 17 (7.8) |
| Abbreviations: NHW, Non-Hispanic White; NHPI, Native Hawaiian and Pacific Islander; CVD, cardiovascular disease | | | | | |
| **Cell value coarsened due to CMS cell size suppression policy. | | |  |  |  |

| *Supplemental Table 3. Sample size, incident cases, and cumulative risk for risk of cardiovascular disease incidence among ANHPI and NHW colorectal cancer survivors diagnosed 2000 to 2017 from SEER-Medicare data, stratified by follow-up period and regional subgroup* | | | | | | | | | | | | | | | |
| --- | --- | --- | --- | --- | --- | --- | --- | --- | --- | --- | --- | --- | --- | --- | --- |
|  | **NHW** | | | **East Asian** | | | **Southeast Asian** | | | **South Asian** | | | **NHPI** | | |
|  | N | Cases | Cumulative Risk (%) | N | Cases | Cumulative Risk (%) | N | Cases | Cumulative Risk (%) | N | Cases | Cumulative Risk (%) | N | Cases | Cumulative Risk (%) |
| All |  |  |  |  |  |  |  |  |  |  |  |  |  |  |  |
| Composite CVD | 4456 | 1290 | 28.9 | 1533 | 385 | 25.1 | 595 | 131 | 22.0 | 90 | 18 | 20.0 | 129 | 27 | 20.9 |
| Heart Failure | 8573 | 1693 | 19.7 | 2356 | 353 | 15.0 | 895 | 124 | 13.9 | 141 | 16 | 11.3 | 182 | 33 | 18.1 |
| Ischemic Heart Disease | 5820 | 1087 | 18.7 | 1845 | 273 | 14.8 | 713 | 113 | 15.8 | 105 | 15 | 14.3 | 152 | 19 | 12.5 |
| Stroke/Transient Ischemic Attack | 10469 | 966 | 9.2 | 2517 | 250 | 9.9 | 1012 | 77 | 7.6 | ** | ** | 7.0 | 193 | 17 | 8.8 |
| >1-5 years |  |  |  |  |  |  |  |  |  |  |  |  |  |  |  |
| Composite CVD | 4456 | 587 | 13.2 | 1533 | 163 | 10.6 | 595 | 82 | 13.8 | 90 | 13 | 14.4 | 129 | 13 | 10.1 |
| Heart Failure | 8573 | 732 | 8.5 | 2356 | 144 | 6.1 | 895 | 70 | 7.8 | ** | ** | 6.4 | 182 | 13 | 7.1 |
| Ischemic Heart Disease | 5820 | 487 | 8.4 | 1845 | 115 | 6.2 | 713 | 72 | 10.1 | ** | ** | 10.5 | ** | ** | 5.9 |
| Stroke/Transient Ischemic Attack | 10469 | 420 | 4.0 | 2517 | 100 | 4.0 | 1012 | 34 | 3.4 | ** | ** | 5.7 | ** | ** | 4.7 |
| >5 years |  |  |  |  |  |  |  |  |  |  |  |  |  |  |  |
| Composite CVD | 1164 | 348 | 29.9 | 567 | 135 | 23.8 | 163 | 30 | 18.4 | ** | ** | 18.8 | ** | ** | 15.2 |
| Heart Failure | 2472 | 478 | 19.3 | 1020 | 152 | 14.9 | 313 | 44 | 14.1 | ** | ** | 10.3 | ** | ** | 17.1 |
| Ischemic Heart Disease | 1572 | 307 | 19.5 | 730 | 103 | 14.1 | 209 | 25 | 12.0 | ** | ** | 15.0 | ** | ** | 10.9 |
| Stroke/Transient Ischemic Attack | 2939 | 257 | 8.7 | 1136 | 106 | 9.3 | 369 | 28 | 7.6 | ** | ** | 0.0 | ** | ** | 8.6 |
| Abbreviations: ANHPI, Asian, Native Hawaiian, and Pacific Islander; CVD, cardiovascular disease; NHW, Non-Hispanic White; NHPI, Native Hawaiian and Pacific Islander | | | | | | | | | | | | | | | |
| N is sample size at risk, excluding prevalent cases | | |  |  |  |  |  |  |  |  |  |  |  |  |  |
| **Cell values suppressed due to the Centers for Medicare & Medicaid Services (CMS) cell size suppression policy. | | | | | | | | | |  |  |  |  |  |  |

| *Supplemental Table 4. Sensitivity analysis for risk of cardiovascular disease incidence among ANHPI and NHW colorectal cancer survivors diagnosed 2000 to 2017 from SEER-Medicare data, stratified by follow-up period and regional subgroup* | | | | | |
| --- | --- | --- | --- | --- | --- |
|  | **NHW** | **East Asian** | **Southeast Asian** | **South Asian** | **NHPI** |
|  | HR (95% CI) | HR (95% CI) | HR (95% CI) | HR (95% CI) | HR (95% CI) |
| All |  |  |  |  |  |
| Composite CVD | Reference | 0.66(0.53,0.82) | 0.99(0.73,1.33) | 0.92(0.46,1.85) | 0.71(0.41,1.24) |
| Heart Failure | Reference | 0.58(0.47,0.71) | 0.85(0.65,1.12) | 0.74(0.36,1.49) | 0.94(0.56,1.58) |
| Ischemic Heart Disease | Reference | 0.67(0.57,0.80)^a^ | 0.95(0.76,1.18)^a^ | 1.28(0.76,2.14)^a^ | 0.70(0.43,1.13)^a^ |
| Stroke/Transient Ischemic Attack | Reference | 0.98(0.76,1.25) | 1.07(0.75,1.52) | 1.06(0.47,2.39) | 0.87(0.45,1.67) |
| >1-5 years |  |  |  |  |  |
| Composite CVD | Reference | 0.78(0.58,1.05) | 1.23(0.85,1.77) | 1.50(0.66,3.45) | 0.91(0.43,1.95) |
| Heart Failure | Reference | 0.67(0.50,0.90) | 1.11(0.78,1.57) | 0.79(0.31,2.01) | 0.96(0.46,2.02) |
| Ischemic Heart Disease | Reference | 0.68(0.48,0.95) | 1.39(0.94,2.05) | 2.52(0.98,6.46) | 0.76(0.32,1.83) |
| Stroke/Transient Ischemic Attack | Reference | 1.15(0.81,1.63) | 1.31(0.81,2.12) | 1.72(0.68,4.35) | 1.14(0.46,2.84) |
| >5 years |  |  |  |  |  |
| Composite CVD | Reference | 0.54(0.36,0.82) | 0.52(0.27,1.00) | 0.25(0.03,2.23) | 0.38(0.13,1.10) |
| Heart Failure | Reference | 0.48(0.34,0.69) | 0.63(0.39,1.02) | 0.86(0.21,3.52) | 0.65(0.26,1.65) |
| Ischemic Heart Disease | Reference | 0.38(0.24,0.62) | 0.30(0.14,0.64) | 1.42(0.18,11.12) | 0.46(0.15,1.36) |
| Stroke/Transient Ischemic Attack | Reference | 0.76(0.48,1.20) | 0.76(0.39,1.48) | ** | 0.60(0.19,1.88) |
| Abbreviations: ANHPI, Asian, Native Hawaiian, and Pacific Islander; CVD, cardiovascular disease; NHW, Non-Hispanic White; NHPI, Native Hawaiian and Pacific Islander; 95% CI, 95% confidence interval | | | | | |
| Models adjusted for SEER registry, income, college education, rural status, baseline Charlson comorbidity index, obesity before cancer diagnosis, and tobacco use disorder before cancer diagnosis. | | | | | |
| ^a^Proportional hazard assumption not met; flexible spline model used. | | |  |  |  |
| **Cell value coarsened due to CMS cell size suppression policy. | | |  |  |  |

| *Supplemental Table 5. Risk of cardiovascular disease incidence among ANHPI colorectal cancer survivors and NHW colorectal cancer survivors for >1 year after cancer diagnosis, stratified by individual ANHPI race and ethnicity groups* | | | | | | | | | |
| --- | --- | --- | --- | --- | --- | --- | --- | --- | --- |
|  |  | **Composite CVD** | | | | **Heart Failure** | | | |
|  |  | N | Cases | HR (95% CI)^a^ | HR (95% CI)^b^ | N | Cases | HR (95% CI)^a^ | HR (95% CI)^b^ |
|  | NHW | 4456 | 1290 | Reference | 1.37(1.01,1.86) | 8573 | 1693 | Reference | 1.77(1.31,2.39) |
|  | Chinese | 575 | 140 | 0.67(0.49,0.91) | 0.92(0.62,1.37) | 960 | 148 | 0.63(0.48,0.82) | 1.11(0.78,1.59) |
|  | Filipino | 352 | 79 | 1.33(0.92,1.92) | 1.83(1.20,2.77) | 526 | 82 | 1.04(0.75,1.44) | 1.84(1.24,2.73) |
|  | Japanese | 712 | 188 | 0.73(0.54,0.99) | Reference | 1002 | 150 | 0.57(0.42,0.76) | Reference |
|  | Vietnamese | 191 | 47 | 0.81(0.51,1.27) | 1.11(0.66,1.87) | 301 | 39 | 0.75(0.49,1.13) | 1.32(0.80,2.16) |
|  | Korean | 246 | 57 | 0.80(0.53,1.22) | 1.11(0.68,1.80) | 394 | 55 | 0.86(0.59,1.25) | 1.52(0.97,2.39) |
|  | Asian Indian and Pakistani | 90 | 18 | 0.85(0.43,1.70) | 1.17(0.56,2.46) | 141 | 16 | 0.83(0.42,1.64) | 1.47(0.71,3.08) |
|  | Native Hawaiian | 94 | 16 | 0.87(0.44,1.74) | 1.20(0.60,2.39) | 131 | 22 | 0.91(0.48,1.72) | 1.61(0.86,3.03) |
|  | Other Pacific Islander^c^ | 35 | ** | 0.58(0.25,1.36) | 0.80(0.33,1.94) | 51 | ** | 0.89(0.38,2.10) | 1.58(0.64,3.88) |
|  |  | **Ischemic Heart Disease** | | | | **Stroke/Transient Ischemic Attack** | | | |
|  |  | N | Cases | HR (95% CI)^a^ | HR (95% CI)^b^ | N | Cases | HR (95% CI)^a^ | HR (95% CI)^b^ |
|  | NHW | 5820 | 1087 | Reference | 1.63(1.16,2.29) | 10469 | 966 | Reference | 0.96(0.67,1.37) |
|  | Chinese | 708 | 107 | 0.75(0.54,1.05) | 1.23(0.80,1.88) | 1042 | 102 | 0.96(0.69,1.33) | 0.92(0.59,1.43) |
|  | Filipino | 423 | 63 | 1.35(0.91,2.01) | 2.20(1.39,3.50) | 622 | 44 | 1.11(0.71,1.71) | 1.06(0.64,1.77) |
|  | Japanese | 840 | 125 | 0.61(0.44,0.86) | Reference | 1049 | 111 | 1.04(0.73,1.49) | Reference |
|  | Vietnamese | 232 | 45 | 0.92(0.57,1.47) | 1.50(0.87,2.60) | 316 | 26 | 0.81(0.45,1.47) | 0.78(0.40,1.53) |
|  | Korean | 297 | 41 | 0.65(0.40,1.04) | 1.06(0.61,1.84) | 426 | 37 | 1.07(0.67,1.71) | 1.02(0.59,1.79) |
|  | Asian Indian and Pakistani | 105 | 15 | 1.59(0.73,3.46) | 2.60(1.12,6.05) | 158 | ** | 1.06(0.47,2.37) | 1.02(0.42,2.44) |
|  | Native Hawaiian | 110 | 12 | 0.85(0.37,1.94) | 1.39(0.62,3.14) | 140 | ** | 0.97(0.40,2.38) | 0.93(0.39,2.24) |
|  | Other Pacific Islander^c^ | 42 | ** | 0.50(0.19,1.30) | 0.81(0.30,2.20) | 53 | ** | 0.81(0.32,2.08) | 0.78(0.29,2.10) |
| Notes: Model adjusted for SEER cancer registry, census-level household income, census-level college education, and rural status. | | | | | | | | | |
| Abbreviations: ANHPI, Asian, Native Hawaiian and Pacific Islander; NHW, Non-Hispanic White; HR, hazard ratio; 95% CI, 95% confidence interval | | | | | | | | | |
| ^a^Model used Non-Hispanic White CRC survivors as reference group | | | | |  |  |  |  |  |
| ^b^Model used Japanese CRC survivors as reference group | | | | |  |  |  |  |  |
| ^c^Includes Micronesian, Chamorran, Guamanian, Polynesian, Tahitian, Samoan, Tongan, Melanesian, Fiji Islander, New Guinean, and Pacific Islander, not otherwise specified | | | | | | | | | |
| **Cell values suppressed due to the Centers for Medicare & Medicaid Services (CMS) cell size suppression policy. | | | | | | | | |  |
